# Efficient Translation of Dnmt1 Requires Cytoplasmic Polyadenylation and Musashi Binding Elements

**DOI:** 10.1371/journal.pone.0088385

**Published:** 2014-02-20

**Authors:** Charlotte E. Rutledge, Ho-Tak Lau, Hazel Mangan, Linda L. Hardy, Olaf Sunnotel, Fan Guo, Angus M. MacNicol, Colum P. Walsh, Diane J. Lees-Murdock

**Affiliations:** 1 Transcriptional Regulation and Epigenetics Research Group, School of Biomedical Sciences, University of Ulster, Coleraine, North Ireland, United Kingdom; 2 Department of Neurobiology and Developmental Sciences, University of Arkansas for Medical Sciences, Little Rock, Arkansas, United States of America; 3 The State Key Laboratory of Molecular Biology, Institute of Biochemistry and Cell Biology, Shanghai Institutes for Biological Sciences, Chinese Academy of Sciences, Shanghai, China; Queen’s University Belfast, United Kingdom

## Abstract

Regulation of DNMT1 is critical for epigenetic control of many genes and for genome stability. Using phylogenetic analysis we characterized a block of 27 nucleotides in the 3′UTR of *Dnmt1* mRNA identical between humans and *Xenopus* and investigated the role of the individual elements contained within it. This region contains a cytoplasmic polyadenylation element (CPE) and a Musashi binding element (MBE), with CPE binding protein 1 (CPEB1) known to bind to the former in mouse oocytes. The presence of these elements usually indicates translational control by elongation and shortening of the poly(A) tail in the cytoplasm of the oocyte and in some somatic cell types. We demonstrate for the first time cytoplasmic polyadenylation of *Dnmt1* during periods of oocyte growth in mouse and during oocyte activation in *Xenopus.* Furthermore we show by RNA immunoprecipitation that Musashi1 (MSI1) binds to the MBE and that this element is required for polyadenylation in oocytes. As well as a role in oocytes, site-directed mutagenesis and reporter assays confirm that mutation of either the MBE or CPE reduce DNMT1 translation in somatic cells, but likely act in the same pathway: deletion of the whole conserved region has more severe effects on translation in both ES and differentiated cells. In adult cells lacking MSI1 there is a greater dependency on the CPE, with depletion of CPEB1 or CPEB4 by RNAi resulting in substantially reduced levels of endogenous DNMT1 protein and concurrent upregulation of the well characterised CPEB target mRNA cyclin B1. Our findings demonstrate that CPE- and MBE-mediated translation regulate DNMT1 expression, representing a novel mechanism of post-transcriptional control for this gene.

## Introduction

Maternal stores of DNMT1 mRNA and protein, accumulated in the egg during oogenesis in vertebrates, are responsible for maintenance methylation in the early embryo, which is reliant on these stores prior to the handover of developmental control to the zygotic genome in the maternal-to-zygotic transition (MZT). A special isoform of DNMT1 is expressed only in oocytes (DNMT1^o^), transcribed from a unique 5′ exon, and is more stable than the isoform expressed in somatic cells (DNMT1^s^) [1]. The maternal stores of DNMT1^o^ appear to be sufficient to allow progression to the blastocyst stage in mouse. DNA methylation in mammalian oocytes is important for the regulation of imprinted genes, disruption of which causes several human disease syndromes [2]. Imprinted genes are active from only one parental chromosome, either the paternal or maternal allele, and the alleles show differential DNA methylation. In most cases, the methylation mark is acquired in the oocyte, with sperm showing no methylation. Deletion specifically of DNMT1^o^ in mouse oocytes causes loss of genomic imprints in offspring and the post-implantation death of resulting embryos [3], [4].

Recent genome-wide studies have found that in fact there are a large number of non-imprinted genes which also acquire maternal-specific methylation in the oocyte and maintain this at relatively high levels through to implantation [5], [6], suggesting that DNMT1^o^ is also important for maintaining methylation at these loci, which may be important developmentally. In non-mammalian systems, DNMT1 also appears to play an important role in early development. Although *Xenopus* lacks imprinting, DNMT1 is required to ensure transcriptional silencing prior to activation of the zygotic genome which occurs at the midblastula transition in *Xenopus* embryos [7].

Mouse ES cells, which are derived from the inner cell mass of the blastocyst, express high levels of the somatic form DNMT1^s^. While ES cells appear to be able to survive in the absence of any DNA methylation, cells lacking DNMT1 quickly die following differentiation [8]. Likewise genetic reduction or ablation in adult differentiated cells triggers the DNA damage response and results in eventual cell death in both cancer cells and in normal hTERT-immortalised cells [9], [10], demonstrating the requirement for the somatic form of the enzyme as well.

We previously identified a consensus cytoplasmic polyadenylation element in mouse, rat and human (UUUUAU) in the *Dnmt1* 3′UTR common to both oocyte and somatic forms of the protein [11]. CPE sequences interact with CPE-binding proteins such as CPEB1 and can direct either repression or activation of target mRNA translation depending on the cellular context. Specifically, while exerting repression in immature, germinal vesicle positive oocytes, CPEs and CPEB1 can direct cytoplasmic polyadenylation and translational activation during *Xenopus*, mouse and human oocyte maturation [12], [13], [14], [15], [16], [17]. CPE-directed mRNA translational control has also been reported in some mouse and human somatic cell types [18], [19], [20], [21]. A second cis-acting sequence must also be present in the 3′UTR for CPE-directed translational activation, termed the polyadenylation hexanucleotide (Hex; AAUAAA), which interacts with cleavage and polyadenylation specific factor (CPSF) for regulation of polyadenylation [22]. CPEB1 activation in mouse oocytes occurs at several discrete points in development, including pachytene of meiosis I, [23] and at the MI to MII transition for translation of mRNAs such as *Cyclin B1* [12]. Additionally, CPEB1 immunoprecipitates with of a range of transcripts at various timepoints throughout oocyte growth, suggesting that it may be active throughout oogenesis [24]. These mRNAs include *Gdf9* and *Dnmt1*, which are highly expressed in growing dictyate oocytes, but a role for CPEB in control of the latter has not been demonstrated.

Recent studies have revealed that mRNA translational control is a highly complex process and typically involves multiple distinct elements that must be functionally integrated [25]. During *Xenopus* oocyte maturation, Musashi binding element (MBE)-dependent control is also crucial for the correct temporal activation of maternal mRNAs. Musashi function is necessary for a subset of maternal mRNAs prior to completion of meiosis I and for the subsequent activation of CPE-dependent mRNA translation [26], [27], [28]. This requirement for translational activation of MBE target mRNAs is in contrast to the well characterized repressive role of Musashi in proliferating somatic stem cells [29]. However, a reconciliation of these functional differences was demonstrated by the context-dependent regulation of translation for MBE-containing mRNA during the transition from neural stem cell proliferation to differentiation. Under these conditions, Musashi switched from a repressor of translation in proliferating stem cells to an activator of target mRNA translation in differentiating cells [30].

Here our aims were to investigate the function of the highly conserved region in the *Dnmt1* 3′UTR in regulating its expression and to begin to characterise the factors which could influence this process. We have extended our phylogenetic analysis of the *Dnmt1* 3′UTR, identifying that the conserved block is preserved as far as zebrafish and also contains a regulatory RNA binding motif for the Musashi protein family in addition to the CPE. We demonstrate that MSI1 expression peaks in growing mouse oocytes, correlating with *Dnmt1* polyadenylation, and that MSI interacts with endogenous *Dnmt1* mRNA by RNA-immunoprecipitation. We also show that *Dnmt1* undergoes dynamic changes in its poly(A) tail length on oocyte growth in mouse and oocyte maturation in *Xenopus*. Early polyadenylation of the murine *Dnmt1* 3′UTR in *Xenopus* oocytes requires the MBE but not the CPE. Site- directed mutagenesis and reporter assays show that in mouse ES cells containing both CPEB and MSI family members, redundant control ensures DNMT1 translation. Human somatic cells lacking MSI1 show greater dependence on CPEB proteins with depletion of either CPEB1 or CPEB4 by RNAi preventing efficient translation of endogenous DNMT1 protein.

## Materials and Methods

### Ethics Statement

All animal experiments were licensed by the UK Home Office in accordance with the UK animals (Scientific Procedures) Act 1986 under Project Licence 2652.

### Sequence Alignments

The human and mouse *Dnmt1* 3′UTR were previously identified from RefSeq genes using BLAT (http://genome.ucsc.edu/cgi-bin/hgBlat) (11). Additional *Dnmt1* 3′UTR sequences for rat (NM_053354), chick (NM_0010407), *Xenopus* (NM_203562) and zebrafish (NM_131189) were identified in the same way. Sequences were compared using ClustalX software [31].

### Mice

Natural matings of Tuck-Ordinary (TO) mice (Harlan, Huntingdon, UK) were used to produce pregnant females. The day the plug was observed was taken as embryonic day 0.5 (e0.5). Animals were housed in a temperature controlled holding room (21.5°C ±1) with a 12∶12 h light cycle. Food and water was available *ad libitum.* Animals were sacrificed by lethal inhalation of CO_2._ Ovaries were dissected from mice of various ages and rinsed in PBS prior to RNA extraction.

### Poly(A) Analysis

**Figure 1:**
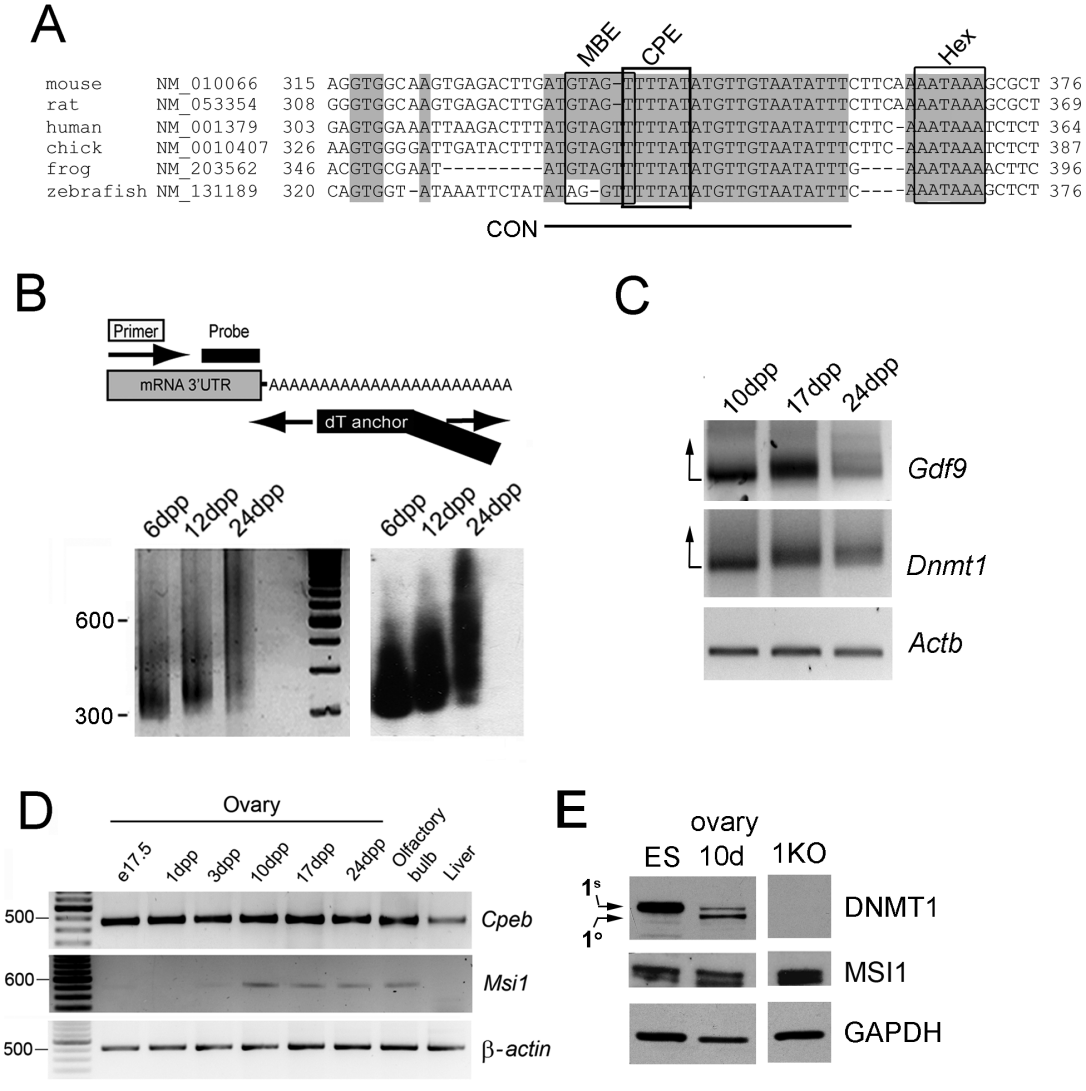
Polyadenylation of *Dnmt1* in the mouse ovary. (**A**) Phylogenetic analysis of the *Dnmt1* 3′UTR in Clustal X shows that the CPE and MBE sites form part of a larger conserved element (CON) which is identical across diverse species. Grey shading denotes areas of 100% homology between species. The CPEB binding site (CPE: UUUUAU in mRNA) is boxed by a thick black line; the consensus RNA binding sequence for mammalian Musashi (MBE; G/AU_1–3_AGU), is present as GTAGT (fine black line). The Hex sequence is also indicated. Species identity and RefSeq accession numbers are indicated at left; Hex- polyA hexanucleotide; CON- conserved region. (**B**) Rapid Amplification of cDNA End-Poly(A) Test (RACE-PAT) to assess polyadenylation levels. *Top panel:* Synthesis of cDNA was carried out with oligo dT primer, and subsequent PCR used a *Dnmt1* gene-specific primer and anchored oligo dT to prevent artificial shortening of the PCR products in subsequent cycles. *Bottom left panel:* Endogenous *Dnmt1* mRNA undergoes polyadenylation in ovaries where the first wave of oocytes are in the growth phase (6–24 days post partum-dpp). *Bottom right panel*: Southern blotting and hybridization with the DNMT1-specific probe indicated above. (**C**) RNA ligation-coupled RT-PCR was used to confirm polyadenylation. Following ligation of an oligo to the poly(A) tails of all mRNA species present in oocytes harvested from ovaries of the indicated age, RT-PCR was carried out using the gene-specific forward primers indicated at right and a primer anti-sense to the ligated oligo. For the *Gdf9* positive control, more of the signal is dispersed upwards on the gel (direction of arrow) as mRNA tails become longer in growing oocytes. *Dnmt1* mRNA can be seen to follow a similar pattern. Control PCR using gene-specific primers for β-actin demonstrates RNA integrity. (**D**) RT-PCR shows that transcripts of *Cpeb1* are present at high levels in ovaries throughout postnatal development, while *Msi1* is expressed in growing oocytes only (10–24 dpp). (**E**) Western blotting shows that only DNMT1^s^ is found in ES cells, whereas DNMT1^o^ begins to appear in growing oocytes and accumulates by 10 dpp. MSI1 shows strongest expression at 10 dpp. Signal for DNMT1^o^ is visible in 3 month old (3 m) adult ovaries on longer exposure. Knockout ES cells lacking DNMT1 (1KO) and GAPDH are shown as controls.

RNA was extracted from mouse ovary using the RNeasy® Mini kit (Qiagen, Crawley, UK), as per the manufacturer’s instructions. RNA concentration was determined using the Nanodrop-1000 spectrophotometer. The RNA-ligation coupled poly(A) test was performed as previously described [32], [33]. Briefly, 100 pmol of oligo linker P1 (5′-GGTCACCTTGATCTGAAGC-3′) was ligated to 500 ng of total RNA using T4 RNA ligase I (New England Biolabs, Hitchin, UK) at 37°C for 30 min. T4 RNA ligase was deactivated by heating to 65°C for 15 minutes. Reverse transcription was performed using Superscript II (Invitrogen, Paisley, UK) with 100 pmol primer P2 (5′-GCTTCAGATCAAGGTGACCTTTTT-3′) in a 50 µl reaction, according to manufacturer’s instructions. For the PCR reaction, the cDNA was subjected to 39 cycles of 30 s at 94°C, 56°C for 1 min and 72°C for 1 min. Each 25 µl PCR contained 1 µl of cDNA, 50 µM of a gene specific forward primer (see Table S1), 50 µM of primer P2, 1x PCR buffer, 1.5 mM of MgCl_2_, 0.5 mM of dNTPs and 0.1 U of Taq polymerase (Invitrogen). Polyadenylation in mouse ovary was also assayed using rapid amplification of cDNA ends- polyadenylation test (RACE-PAT) as described previously [34]. Briefly 200 ng of RNA was reverse transcribed using an anchored oligo(dT) primer (5′ GCGAGCTCCGCGGCCGCGT_12_) as above. PCR was subsequently carried out as above using a *Dnmt1* gene-specific forward primer (see Table S1) and anchored oligo(dT) reverse primer. PCR conditions were 94°C for 5 min followed by 35 cycles of 91°C for 30 sec, 60°C for 1 min and 72°C for 1 min and a final elongation at 72°C for 7 min. PCR products from both methods of poly(A) analysis were electrophoresed on a 3% agarose gel. RACE-PAT products were Southern blotted, radiolabelled probe generated and hybridisation performed as previously described [35]. The radiolabelled probe (5′-GACAGAAGCGCTTTATTTTGAAGAAATATTACAACATATAAAACT ACATCAAG-3′) incorporated the CPE and Hex sites and was located as depicted in Fig. 1B. RNA-ligation coupled poly(A) test was performed on *Xenopus* oocytes as previously described [36], and products were directly sequenced to confirm product identity.

### RT-PCR and RT-qPCR

RNA was extracted from oocytes, tissues and cells as above. cDNA synthesis was carried out using Superscript II reverse transcriptase (Invitrogen) with 500 ng RNA, 500 ng oligo (dT)_15_ primer, 0.5 mM dNTPs, 1x first-strand buffer and 5 mM DTT according to manufacturer’s instructions. RT-PCR was performed in 25 µl reactions containing 0.5 µM of each primer (see Table S2), 3.7 mM MgCl_2_, 0.4 mM dNTPs, 1x PCR buffer, 0.1 U Taq polymerase and 1 µl cDNA. Initial denaturation of 94°C for 3 min was followed by 35 cycles (25 cycles for *β-actin*) of 91°C for 30 s, gene-specific annealing temperature (see Table S2) for 30 s, and 72°C for 1 min with a final elongation of 72°C for 3 min. RT-qPCR was carried out on the LightCycler® 480 II (Roche, West Sussex, UK). Standard curves were conducted for all primer sets to determine primer efficiency. Melting curves were checked to determine the presence of a single, sharp peak, and saturated PCR products were run on a 1% agarose gel and visualised by UV transillumination to ensure amplification of a single product of correct size. Subsequently reactions were set up in triplicate for the gene of interest and for a housekeeping gene for each sample (see Table S3), using LightCycler® 480 SYBR Green I Master (Roche), as per manufacturer’s instructions. Reactions were run on the LightCycler® 480 II, with an initial incubation step of 95°C for 10 min, followed by 50 cycles of 95°C for 10 s, 60°C for 10 s and 72°C for 10 s, with a final ramping of the temperature to 97°C to generate the melting curve. Following completion of the programme relative expression levels were calculated in the LightCycler® 480 Software release 1.5.0 SP3 (Roche). The results over three independent experiments were averaged, and the Microsoft Excel t-test function (two-tailed, unpaired) was used to determine statistical significance.

### Cell Culture

HeLa (ATCC) and HCT116 [37] (kind gift of R. Farber) cells were maintained in 1 g/L glucose D-MEM (Invitrogen), both supplemented with 10% FBS (Invitrogen) and 1x NEAA (Invitrogen). R63 ES cells [38] (kind gift of H. Wheadon) were maintained on Nunc tissue culture plates (Davidson & Hardy Ltd., Belfast, UK) which were gelatinised with 0.1% porcine gelatin (Sigma-Aldrich, Dorset, UK). Components of ES cell medium are from Invitrogen unless otherwise stated. ES cells were cultured in KnockOut™ D-MEM supplemented with 15% KnockOut™ Serum Replacement, 1% ES-cell qualified fetal bovine serum, 1x NEAA, 2 mM L-glutamine and 0.1 mM β-mercaptoethanol (Sigma-Aldrich). Stable CPEB1 knockdown cells were created using the Knockout™ Single Vector (Clontech, Sainte-Germaine-en-Laye, France) as per the manufacturer’s instructions, and were maintained under the same culture conditions as the parental HeLa cells with the addition of 400 µg/µl geneticin (Invitrogen). All cells were cultured at 37°C, 5% CO_2_ in a humidified atmosphere.

### Western Blotting

Cells were collected, washed in PBS and spun at 200×g for 5 min at 4°C. PBS was removed and 50 µl of protein extraction buffer (50 mM Tris-HCl, 150 mM NaCl, 1% Triton-X, 10% glycerol, 5 mM EDTA) +0.5 µl protease inhibitor cocktail for mammalian cells (Sigma-Aldrich) was added. Solution was incubated on ice for 20 min. Subsequently, lysate was spun at 16,100×g for 10 min at 4°C. Protein concentration was determined by Lowry assay, and samples were kept at −80°C until use. SDS-PAGE was used to resolve 30 µg of protein from each cell line, followed by electroblotting onto a nitrocellulose membrane (Amersham Biosciences, Amersham, UK), which was then blocked overnight at 4°C in 5% non-fat dried milk. Membrane was incubated with primary antibody (Table S4) before incubation with horse radish peroxidise (HRP)-coupled secondary. All incubations were carried out for 1 hr at room temperature. Detection was carried out with ECL (Amersham Biosciences).

### Reporter Vectors

The *Dnmt1* 3′UTR was amplified by PCR from mouse tail DNA. The forward primer was designed to carry an *Xba*I restriction site (5′-GTAGCTCTAGACCATCATTTGA AGTCTTGTGC-3′) and the reverse primer carried a *BamH*I restriction site (for WT and ΔCPE, 5′-GATTCGGATCCGAAGCG CTTTATTTTGAAGAA-3′; for ΔHEX, 5′-GATTCGGATCCGAAGCGCTGGATTTTGAAGAA-3′; for ΔCON, 5′-GATTCG G ATCCGGGTGCTTGACAGAAGCGCTTTATTTTGAAGCAAGTCT CACTTGC -3′). The PCR products were double digested with *Xba*I (Invitrogen) and *BamH*I (Invitrogen) and were inserted into the *Xba*I/*BamH*I digested pGL3 promoter plasmid (Promega, Southampton, UK), replacing the SV40 late poly(A) signal. Ligations were carried out overnight at 16°C with T4 DNA ligase (Invitrogen). ΔMBE and ΔCΔM constructs were created from the WT reporter construct using the QuikChange® Lightning Site-Directed Mutagenesis kit (Stratagene, Dublin, Ireland). Primers were designed according to manufacturer’s specifications to be fully overlapping, with the reverse primer sequence exactly corresponding to the reverse complement of the forward primer. Forward muatagenesis primer sequences are as follows- ΔMBE- 5′-GCAGGTGGCAAGTGAGACTTGATGTAGCCCT TTTATAT GTTG-3′; ΔCΔM- 5′-GCAGGTGGCAA GTGAGACTTGAT GTAGCCCTCCCAT ATGTTG-3′. The mutagenesis reaction was carried out as per the manufacturer’s instructions.

### Luciferase Assays

Cells were seeded at 2.5×10^4^ per well in a 96-well plate and incubated overnight. Two hundred nanograms of wild type or mutant *Dnmt1* 3′UTR-pGL3 plasmid and 20 ng of pRL-TK plasmid (Promega) were transfected into the cells by incubating with 0.75 µl *Lipofectamine*™ 2000 (Invitrogen) per well for 6 hours in serum free Opti-MEM (Invitrogen) according to manufacturer’s instructions. Transfected cells were incubated overnight in complete medium. Cells were used for either luciferase assay or RT-PCR. Luciferase assay was conducted using the Dual*-*Luciferase*®* Reporter Assay Kit (Promega) and a FLUOstar Omega luminometer (BMG Labtech, Aylesbury, UK), according to the manufacturer’s instructions. The firefly luciferase readings were normalized to the Renilla luciferase readings. Results are representative of triplicate samples from more than three independent experiments and are presented as mean +/− standard error of the mean (SEM). Data groups were compared using the unpaired Student’s *t* test.

### RNA-Immunoprecipitation (RIP)

RIP analysis was performed using the Magna RIP™ RNA-Binding Protein (RBP) Immunoprecipitation Kit (Cat. # 17–700, Merck KGaA, Darmstadt, Germany), according to manufacturer’s instructions. Briefly, 5 µg of antibody (MSI1 and IgG; Cat. # 03–114, Merck KGaA) was bound to magnetic beads and RBP’s were immunoprecipitated from R63 cell lysate for 6 hours. Proteins were digested and total RNA was extracted using the RNeasy kit as described above. RT-PCR and RT-qPCR analysis (absolute quantification) were performed as described previously using primers outlined in Table S5. RIP results are representative of triplicate samples of two independent experiments and are presented as mean +/− sd. Data groups were compared using the unpaired Student’s *t* test.

## Results

### A Conserved Region in the *Dnmt1* 3′UTR Includes a CPE and MBE

We previously identified a cytoplasmic polyadenylation element (CPE) in the 3′UTR of the *Dnmt1* gene which is conserved between human and mouse (11). To further investigate possible sequence elements in this region, we used Clustal X software to align the RefSeq DNA sequences from various different species and clades. This revealed a block of 27 nucleotides containing the CPE which shows total conservation across a diverse range of species including mouse, rat, human, chicken and frog, and which differs at only 2 nucleotides in zebrafish (Fig. 1A, underlined). Not only is the CPE present in these species but we also found a binding site for the sequence-specific RNA binding proteins of the Musashi family. The consensus RNA binding sequence for mammalian Musashi, G/AU_1–3_AGU [39], is present as GTAGT in the DNA sequence of the *Dnmt1* 3′UTR. The sequence and location of the *Dnmt1* poly(A) hexanucleotide is also well conserved among these species. The almost complete conservation of this region, even in species which do not have imprinting such as frog, suggests an important role in the oocyte and/or somatic cell functions of DNMT1.

### Polyadenylation of *Dnmt1* in Oocytes

To investigate whether *Dnmt1* mRNA undergoes CPE- or MBE-mediated polyadenylation in oocytes we employed rapid amplification of cDNA- poly(A) test (RACE-PAT) on ovaries from neonatal mice. Anchored oligod(T) primer was used for cDNA synthesis followed by amplification with a gene-specific forward primer and the anchored oligo to prevent artificial shortening of the PCR products in subsequent cycles (Fig. 1B, top panel). This method confirmed that as the first wave of oocyte growth occured between 6 and 24 days post partum (dpp) in mice, *Dnmt1* mRNAs showed progressively more elongated poly(A) tails (Fig. 1B, bottom left panel). To confirm the identity of the amplification products and highlight changes in transcript length, DNA was transferred to a nylon membrane and hybridized to a probe specific for the *Dnmt1* 3′UTR but which, importantly, did not overlap either primer used for the RACE-PAT (Fig. 1B, right panel). Fig. 1B (bottom right) shows clearly that the RACE-PAT products of different sizes contain the probe region and, since the 5′ primer is in a fixed position, the heterogenous sizes seen in Fig. 1B must be due to variation at the 3′ end of the RACE-PAT products.

We used a second method, RNA ligation-coupled RT-PCR to confirm this result. An oligo was ligated to the end of the poly(A) tails of mRNA isolated from ovaries, the mRNA were then reverse-transcribed using an antisense oligo, prior to PCR with a gene-specific forward primer. *Gdf9*, which interacts with CPEB1 in growing mouse oocytes to mediate its polyadenylation [24], was used as a positive control and displayed increasing poly(A) tail length in ovaries from 10 to 24 dpp mice as expected (Fig. 1C, top panel). The pattern of gel retardation of *Dnmt1* PCR products is similar, confirming poly(A) tail elongation occurring at this stage as seen with RACE-PAT. A control PCR using both forward and reverse gene-specific primers for β-*actin* demonstrates the integrity of the RNA.

To examine the relative timing of expression of MSI1, CPEB1 and DNMT1 we first carried out RT-PCR in mouse ovaries from a range of developmental time-points during oogenesis (Fig. 1D). While *Cpeb1* transcripts were expressed at all stages examined in accordance with previous studies [14], [23], [40], MSI1 was found only in ovaries containing growing oocytes (10 dpp onwards). We confirmed strong MSI1 expression by western blotting at 10 dpp, with levels decreasing as ovaries mature (Fig. 1E). DNMT1 has two isoforms, an oocyte-specific form DNMT1^o^ and a somatic form DNMT1^s^ (see above), which differ by a short stretch of AA at the amino-terminus. Using an antibody which detected both the oocyte-specific and somatic forms of the protein, mouse ES cells were found, as expected, to contain only the somatic form of the protein [41]. DNMT1^o^ protein appears in growing oocytes from 10 dpp onwards as we and others have previously shown [42], [43]. The band corresponding to DNMT1^s^ in these samples reflects DNMT1^s^ from somatic cells of the ovary, where transcripts are present at 1000-fold lower levels than in oocytes. Protein from *Dnmt1* knockout ES cells was used as a negative control [43].

### Musashi Plays a Key Role in Polyadenylation in Xenopus Oocytes

**Figure 2:**
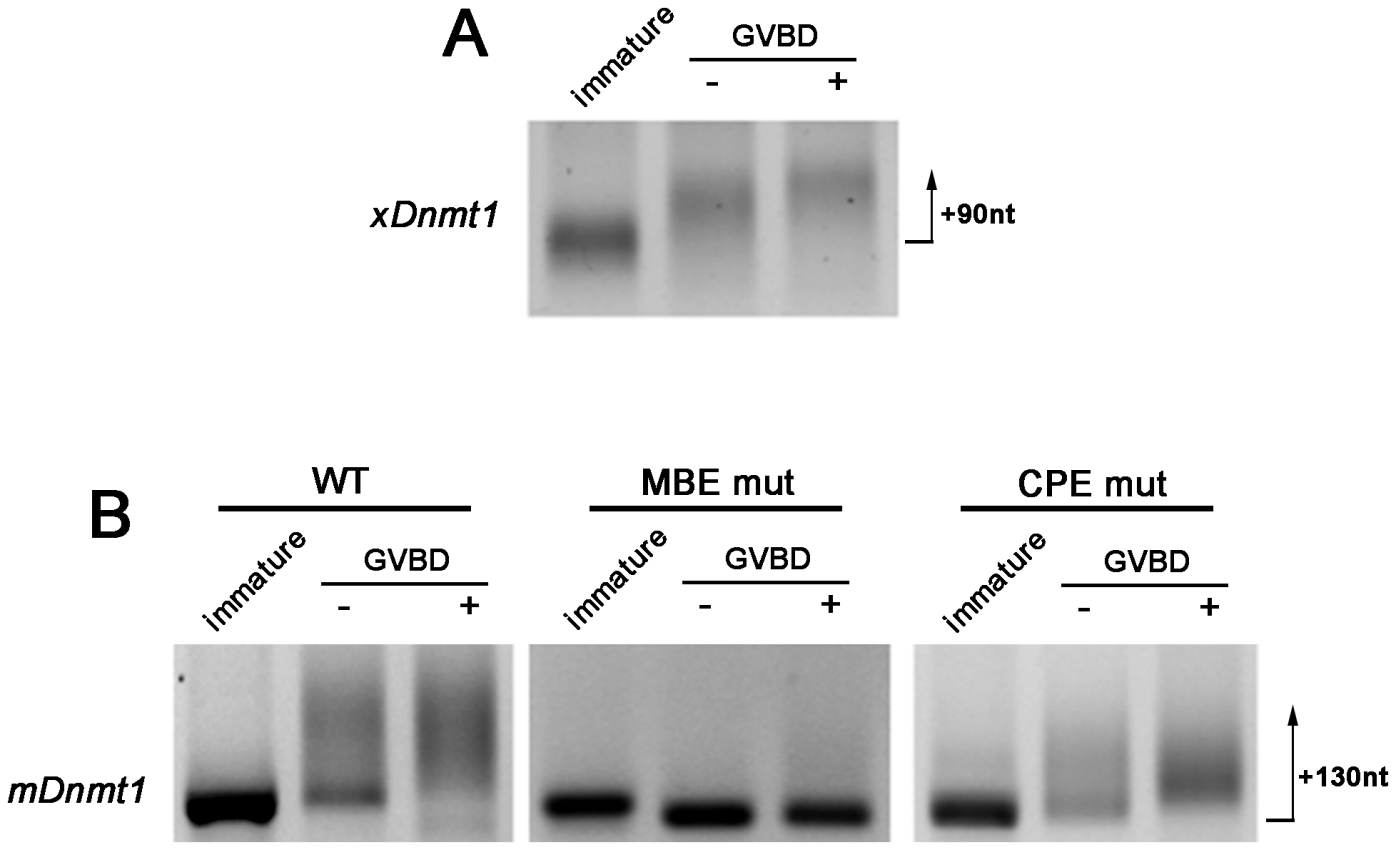
Msi plays a key role in polyadenylation in the *Xenopus* oocyte. (**A**) Polyadenylation occurs at GVBD in *Xenopus* oocytes. RNA ligation-coupled RT-PCR indicates that the endogenous *Xenopus Dnmt1* mRNA (*xDnmt1*) has a short poly(A) tail in immature oocytes, but becomes elongated during progesterone stimulated maturation (arrow indicates extent of polyadenylation). Oocytes were collected when 50% had undergone germinal vesicle breakdown (GVBD) and grouped into those which had (+) or had not (−) completed GVBD. The maximum size of poly(A) tail confirmed by sequencing is indicated in nucleotides (nt) beside the arrow. (**B**) Polyadenylation of mouse *Dnmt1* (*mDnmt1*) in *Xenopus* oocytes requires the MBE but not CPE. In vitro transcribed wildtype (WT), MBE mutant (MBE mut), or CPE mutant (CPE mut) *Dnmt1* 3′UTR constructs were injected into immature *Xenopus* oocytes, which were then stimulated with progesterone treatment and assayed as above. When the MBE is mutated, polyadenylation does not occur, while the pattern of polyadenylation of the CPE mutant is similar to wildtype. Max confirmed poly(A) tail size by cloning indicated as for (A).

We wished to test directly whether the CPE or MBE were important in polyadenylation. Due to the small size of the mouse oocyte and limiting material, this is not normally possible in this system, so we turned to *Xenopus*, which retains the conserved region of the 3′UTR (Fig. 1A) and which has oocytes orders of magnitude larger. Initially we used RNA-ligation coupled RT-PCR to demonstrate that the endogenous *Xenopus Dnmt1* mRNA undergoes progesterone-dependent polyadenylation (Fig. 2A). *Dnmt1* polyadenylation appears to be an early event occurring in mature eggs just prior to completion of germinal vesicle breakdown (GVBD), the name given to the disintegration of the nuclear membrane prior to entry into Meiosis I. Direct sequencing confirmed the identity of the *Xenopus Dnmt1* PCR products and determined poly(A) tail length to be up to 90 nucleotides (Fig. 2A).

To assess whether the mouse *Dnmt1* mRNA can also be polyadenylated by the *Xenopus* oocyte machinery, we microinjected the wild-type murine *Dnmt1* 3′ UTR into *Xenopus* eggs: polyadenylation of the mouse construct was faithfully replicated just prior to GVBD (Fig. 2B, left panel), consistent with the endogenous *Xenopus* mRNA (Fig. 2A). Direct sequencing confirmed the identity of the PCR products and determined poly(A) tail lengths of up to 130 nucleotides were present.

To determine whether the CPE or MBE were important for poly(A) addition, we generated mouse *Dnmt1* 3′ UTR constructs containing a mutationally disrupted MBE or CPE and injected these into immature *Xenopus* oocytes as before. A construct containing a previously-validated loss of function mutation in the MBE site (GTAGT = >GccGT, [26]) but which retained an intact CPE was not polyadenylated prior to GVBD in maturing oocytes (Fig. 2B, middle panel). In contrast, mutational disruption of the CPE did not prevent polyadenylation, although a tendency towards decreased *Dnmt1* mRNA poly(A) tail was noted (Fig. 2B, right panel). The results in oocyte systems overall suggest that cytoplasmic polyadenylation of the *Dnmt1* mRNA at this stage in development is likely to require Musashi function and an MBE in the *Dnmt1* 3′ UTR.

### Redundant Control Involving MSI1 in Mouse Embryonic Stem Cells

**Figure 3:**
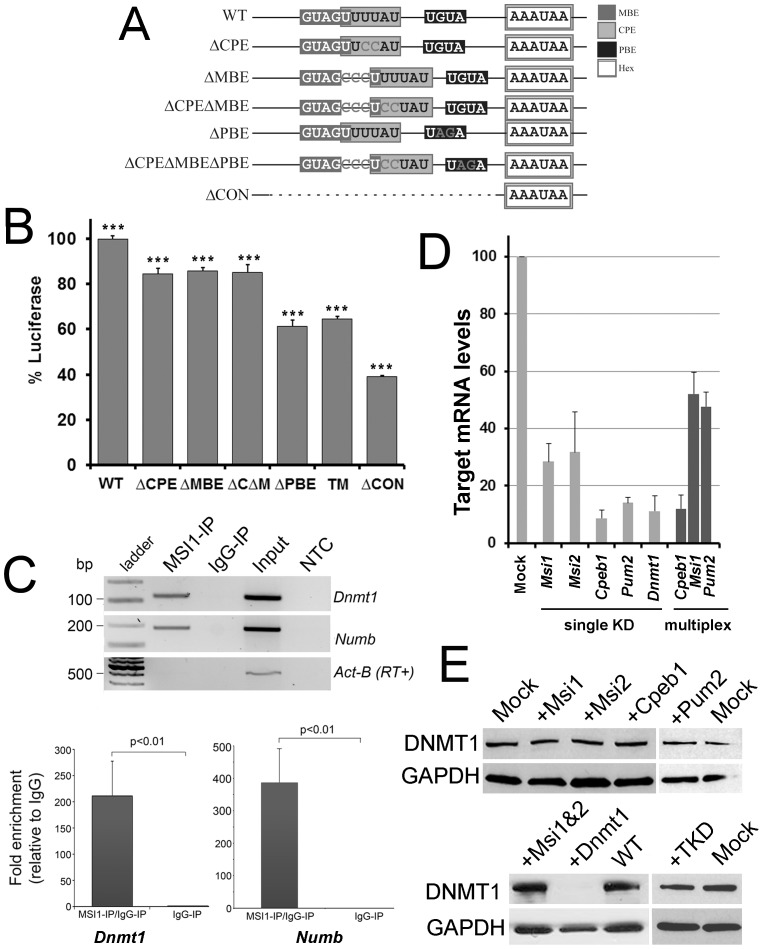
Redundant control involving MSI and CPEin mouse embryonic stem cells. (A) Schematic to show the mutations used to test functional requirements in the *Dnmt1* 3′UTR after cloning it downstream of a luciferase reporter. As well as the CPE and MBE, the region contains a potential Pumilio binding element (PBE): all three were mutated (dark grey letters) using point mutation or insertions (MBE). The ΔCON construct contains a deletion of the entire conserved block indicated in Fig. 1. (**B**) Mutations in the CPE (ΔCPE) MBE (ΔMBE), or PBE significantly decrease translation of the luciferase reporter in mouse ES cells, but combined mutations (ΔCΔM; or triple mutant, TM) do not have an additive effect. Deletion of the conserved region reduces translation to below 40% of the wildtype *Dnmt1* 3′UTR. Readings in triplicate from more than three independent experiments are presented as mean +/−SEM; ***p<0.001 (by 2-tailed unpaired Students T-test) compared to expression of wildtype, which was arbitrarily set to 100%. (**C**) MSI1 interacts with endogenous *Dnmt1* mRNA. RNA-immunoprecipitation was performed in R63 mouse ES cells with anti-MSI1 antibody or normal rabbit IgG. Immunoprecipitated RNA was analysed by RT-PCR (top panel). The known MSI1 target *Numb* could be amplified, while *Actb* which is not a target, was absent, confirming the technique was working. *Dnmt1* could be amplified from MSI1 immunoprecipitates, but not negative controls, indicating MSI1 also binds to this mRNA. A no template control (NTC) was also included. Quantification by RT-qPCR (bottom panel) indicated significant enrichment of *Dnmt1,* to levels approx. half that of *Numb* (bottom right). Results are the average of three independent experiments carried out in triplicate. (**D**) Transient knockdown of RNA binding proteins in ESC. Levels of depletion of the respective target mRNAs following transfection by the indicated siRNA were assessed by RT-qPCR. A scrambled siRNA (mock) was used as a control. An example multiplex experiment (dark bars) is shown at right. All target mRNA were significantly reduced (p<0.05) in single or multiplex experiments. (**E**) Western blotting showing redundant control ensures DNMT1 translation in ESC. Transient depletion of any one of the RNA binding proteins MSI1, MSI2, CPEB or PUM2, or of combinations thereof such as MSI1 and MSI (MSi1&2) and MSI1, CPEB1 and PUM2 (TKD), had no detectable effect on DNMT1 protein levels in ESC. DNMT1 siRNA are shown as a positive control, mock and WT are negative controls.

While stores of DNMT1 laid down in the egg are sufficient to program development to the stage of implantation, DNMT1^s^ expression is strongly up-regulated around implantation and in ES cells, and is required post-implantation for cells to survive differentiation [8]. ES cells also express high levels of MSI1, MSI2, CPEB1 and CPEB2, so we tested whether the conserved region in the UTR was required for translation in this system as well. We cloned the WT *Dnmt1* 3′UTR into a reporter vector downstream of firefly luciferase to replace the SV40 poly(A) signal. This vector was then used to generate constructs containing mutations in the CPE (ΔCPE) by converting uracil to cytosine, and in the MBE mutants (ΔMBE) by inserting a triplet of cytosines, as indicated in Fig. 3A. A double mutant (ΔCPEΔMBE) and a mutant with the whole conserved region deleted (ΔCON) were created using a similar strategy. Mutation of the CPE or MBE reduced expression of the reporter in mouse ES cells to very similar levels (85% and 86%, respectively: p<0.05; Fig. 3B). The double mutant showed identical activity (85%, p<0.05, indicating that these two mutations are likely to be redundant. However, deletion of the whole conserved region reduced reporter expression to less than 40% of wildtype (p<0.05), indicating that further elements in this region play a role in translation independent of the MBE and CPE. We examined the 3′UTR for any other possible conserved binding sites and uncovered a tetranucleotide Pumilio binding element (PBE) which may be bound by members of the Pumilio family, including PUMILIO2 (Pum2) which is expressed in ES cells. Mutation of the PBE (Fig. 3A) resulted in a greater reduction in luciferase activity than seen for the MBE or CPE (Fig. 3B). However mutation of all three elements (Fig. 3A) still did not reduce activity to the level seen when the conserved region was deleted (Fig. 3B).

While there is already evidence that *Dnmt1* mRNA forms a complex with CPEB1 [24], no Musashi family member has yet been shown to interact with the mRNA. We therefore carried out RNA-IP on lysates from R63 mouse ES cells with an anti-MSI1 monoclonal antibody. Successful immunoprecipitation was confirmed by amplification of *Numb*, a previously validated target [39] by RT-PCR (Fig. 3C, top) and RT-qPCR (Fig. 3C, right panel). *Dnmt1* mRNA was enriched to a similar degree as *Numb* (p<0.01; Fig. 3C, top and left). *ActB*, which does not interact with MSI1, was used as a negative control. Similar experiments with commercially-available PUM2 antibodies failed to immunoprecipitate positive control mRNA.

Given that mutations in the CPE and MBE affect translation and that CPEB [24] and MSI (above) bind to the mRNA, we carried out genetic depletion experiments in R63 ES cells by introducing siRNA targeting *Msi1* or *Cpeb1*. *Dnmt1* siRNA was used as a positive control. Each siRNA was shown to effectively reduce levels of the target mRNA (Fig. 3D). However, depletion had no apparent effect on DNMT1 protein levels, which appear to remain unchanged compared to mock treated controls (Fig. 3E, top panel). As MSI2 and PUM2 are also present in ES cells, we carried out further siRNA treatment of *Msi2* and *pum2* (Fig. 3E top panel) to similar effect. We also tested combinations of siRNA such as MSI1 and MSI2 together (MSI1&2, Fig. 3E, bottom panel) as well as triple knockdown (TKD) of CPEB1, MSI1 and PUM2. Although these multiplex experiments were typically less effective at reducing their target mRNA levels (Fig. 3D), these results on the whole suggest that while the sites and the conserved region are important, there is likely to be redundant control of translation for this vital protein in embryonic stem cells.

### CPEB Proteins Play an Important Role in DNMT1 Regulation in Adult Differentiated Cells

**Figure 4:**
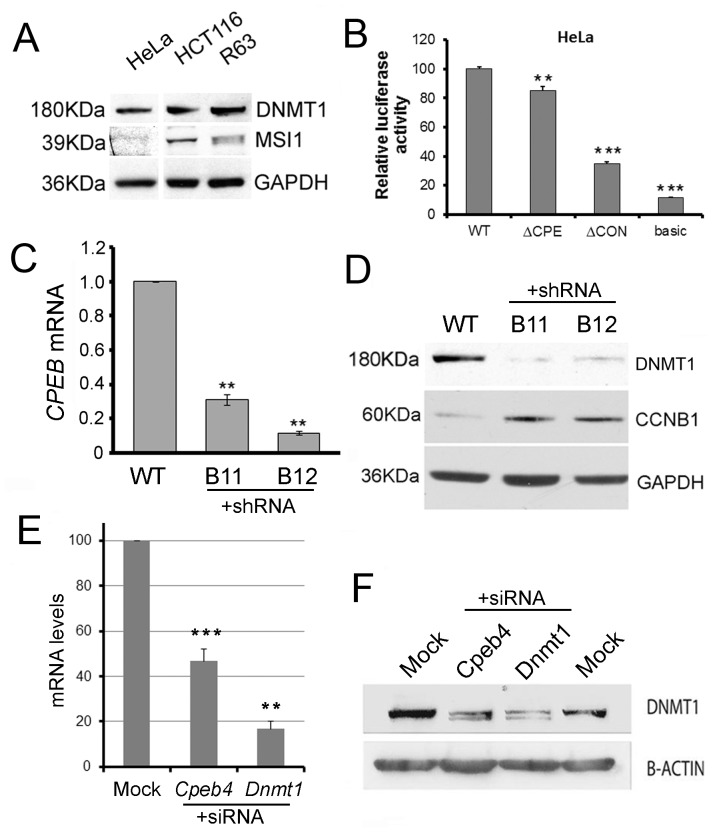
Cells lacking MSI1 show greater CPEB dependence. (**A**) HeLa cells lack MSI1. Western blotting shows that while DNMT1 is expressed in HeLa, HCT116 and R63 ES cells, the expression of MSI1 is limited and detected only in HCT116 and R63 ES cells. Sizes are indicated in kilodaltons (kDa). Similar results were found for PUM2 (**B**) CPE is one important component of translational control in HeLa. Luciferase constructs as indicated in Fig. 3 were transfected into HeLa cells. Mutation of the CPE reduced expression of the reporter gene to a similar extent as observed in ESC cells. Significance **p<0.01; ***p<0.001 (**C**) CPEB1 was targeted for knockdown in HeLa cells by stable shRNA expression. Levels of *CPEB1* mRNA in two clonally-derived cell lines B11 and B12 carrying the shRNA are shown. (**D**) *Dnmt1* mRNA levels remain stable in CPEB1 knockdown cell lines. (**E**) Stable CPEB1 knockdown prevents efficient DNMT1 translation. Protein levels of CCNB1, which is known to be translationally repressed by CPEB1, show upregulation in both stable knockdown cell lines as expected. Reprobing the same membrane shows DNMT1 protein levels are reduced in these samples. Molecular weights are indicated to the left in kiloDaltons (KDa). (**F**) CPEB4 transient knockdown in HeLa. RT-qPCR shows mRNA levels relative to mock-transfected cells following transient knockdown of CPEB4 and DNMT1 in HeLa. (**G**) Transient depletion of CPEB4 reduces DNMT1 protein levels to an extent comparable to that achieved by *DNMT1* siRNA.

While MSI1 is expressed in oocytes and ES cells, it is absent in many adult differentiated cell types where DNMT1 is found, such as HeLa cells (Fig. 4A). However CPEB1 is known to be functionally important in HeLa and some other cell types. We assessed the importance of the CPE, and the conserved region as a whole, for DNMT1 translation in HeLa cells using our luciferase constructs as before (Fig. 4B). Mutation of the CPE led to a significant reduction in luciferase expression (p<0.05; Fig. 4B), while expression was reduced to less than 40% when the whole conserved region was deleted (ΔCR, p<0.05), suggesting that other factors may also contribute to the regulation of Dnmt1. MBE and PBE mutations were not tested given the absence of these proteins in this cell type.

To investigate further the role of CPEB1 in this adult cell type, we generated cell lines carrying stable knockdowns of CPEB1 using short hairpin RNA (shRNA). Two knockdown cell lines, B11 and B12, were chosen for further analysis. RT-qPCR analysis (Fig. 4C) showed clone B11 had 31.5% of wildtype *Cpeb1* mRNA levels (p<0.01), while clone B12 had only 11.7% (p<0.01). *Dnmt1* mRNA levels remained at WT in both clonal lines (Fig. 4D). CPEB can have positive or negative effects on translation depending on the combination of CPE sites, their spacing and the presence or absence of additional RNA binding protein sites [44]. The CPE in the 3′UTR of the cyclin B1 gene (CCNB1) have previously been shown to be important for CPEB-mediated repression of CCNB1 [45] and we found levels of CCNB1 to be greatly increased in both CPEB1 knockdown cell lines as expected demonstrating that CPEB1 levels were reduced sufficiently to dysregulate at least one of its targets. Importantly, and as predicted from the positioning of the CPE site, DNMT1 protein levels were instead reduced in the two knockdown cell lines compared to the wildtype parental cell line (Fig. 4E).

As well as CPEB1, many adult cell types express the related protein CPEB4, which may also bind to CPE present in target mRNA. To explore this possibility, we used short interfering RNA (siRNA) to target *CPEB4*. As a positive control affecting DNMT1 protein levels, siRNA against *DNMT1* were used and gave direct reductions in *DNMT1* target mRNA (Fig. 4F), leading to effects on DNMT1 protein level as expected (Fig. 4G). Treatment of cells with siRNA to *CPEB4* could reduce levels of *CPEB4* mRNA substantially in HeLa cells (Fig. 4E), without affecting DNMT1 mRNA levels (not shown). Western blotting indicated that transient CPEB4 knockdown did however affect *DNMT1* translation (Fig. 4F) in a manner comparable, though less pronounced, than targeting the *DNMT1* mRNA directly.

## Discussion

Such wide conservation among a diverse range of species as seen in the *Dnmt1* 3′UTR is uncommon and signals a conserved role for RNA binding proteins in control of *Dnmt1* expression. To our knowledge, *Dnmt1* is the only mRNA where the CPE and MBE are both conserved from *Xenopus* through to human. While *Xenopus cyclin B5* mRNA 3′UTR contains both CPE and MBE motifs, an orthologue does not exist in mammals. The *Xenopus Mos* proto-oncogene also contains these two elements but the orthologous mammalian *Mos* lacks the MBE [17]. Evolutionary conservation of the CPE and MBE in the *Dnmt1* 3′UTR in organisms which lack imprinting e.g. *Xenopus* and zebrafish, points to a possible role for RNA binding protein-dependent regulation of *Dnmt1* in somatic cells as well as the germline. The CPEB and MSI family members have roles in some adult somatic cell types apart from oocytes [20], [45], [46], [47], [48] and while we cannot rule out an indirect mechanism of action, we have shown here that it is likely that the CPE and MBE in the *Dnmt1* 3′UTR contribute to efficient expression in somatic cell lines.

Recent studies have devised a computational formula for determining the timing and extent of CPE-regulated translation [44], [49]. CPEB can either activate or repress transcription depending on the cofactors it recruits [44] and these models suggest that *Dnmt1* should be translationally activated by the CPE. We found this to indeed be the case, as deletion of the CPE reduced translation of a reporter coupled to the DNMT1 3′UTR in both ES cells and adult differentiated cells, and knockdown of CPEB1 or CPEB4 directly reduced levels of the protein in HeLa cells. However, CPE-containing mRNAs are not solely regulated by CPEB family members and other sequences present in their 3′UTRs also help to modulate the timing of their translation [50], [51], [52]. The MBE in the DNMT1 3′UTR is also totally conserved and we demonstrate that it is bound by MSI1 protein. It is notable that mutation of the MBE gave exactly the same degree of translational repression as disruption of the CPE in ES cells, where both proteins are present, and there was no additive effect when both sites were mutated. This may be because the MBE and CPE sites overlap and thus disruption may be affecting both, or alternatively it may suggest that they are acting in the same pathway.

The experiments in oocytes suggest that MSI acts upstream of CPEB, as mutational disruption of the MBE abrogated early polyadenylation in oocytes, whereas mutation of the CPE did not, though poly(A) tails may have been slightly reduced in length. Similar precedence for MBE function over CPE function has been reported for the translational activation of *Xenopus Mos*, *cyclin B5* and *Musashi1* (Nrp1A/B) mRNAs [26], [27], [28], leading to the proposal that there is a hierarchy of distinct RNA translational control factors [25]. Bearing this out, we found that while the MBE is crucial for polyadenylation in growing oocytes, adult differentiated cells lacking Musashi show strong dependence on CPEB family members instead. The complete conservation of sequences stretching well beyond the CPE and MBE in the *DNMT1* 3′UTR strongly suggest roles of other RNA binding proteins, which also act in a cell-context-specific fashion to modulate translation of the protein. In this context, we found that mutation of a potential PBE in this region independently reduced luciferase activity of our reporter. However mutations in all three elements did not reduce translation to the levels seen in constructs lacking the entire conserved block, either in stem cells (Fig. 3B) or differentiated cells (data not shown). This suggests that other elements in the conserved region are more important in ES cells. Since KD of CPEB1 or CPEB4 alone in HeLa, or mutating the MBE alone in oocytes, was sufficient to give marked effects on DNMT1 translation, there must also be a redundancy in RNA BP usage in ES cells, though we cannot rule out the failure to reduce target mRNA levels sufficiently in multiplex experiments. Such high redundancy in RNA BP binding and control in some situations has been found by others. For example, up-regulation of AUF1 has been suggested as a compensatory mechanism to explain the phenotype of mice lacking MSI proteins [53] and AUF1 has also been reported to regulate DNMT1 by binding to the 3′UTR [54].

Failure to polyadenylate *DNMT1* in oocytes is predicted to have severe consequences for the subsequent zygote. In *Xenopus*, xDNMT1 is necessary for proper control of zygotic gene activation after fertilization [7], [55] and embryos lacking the enzyme fail to develop fully. In mouse too, mutations which remove the oocyte form of the protein cause subsequent death of the embryo when the eggs are fertilised [3] and this was thought to be primarily due to the dysregulation of imprinted gene expression. However recent studies show that, as in *Xenopus*, there are a large number of non-imprinted genes which inherit methylation from the egg and retain methylation up until the blastocyst stage [5], [6]. Maternal stores of DNMT1^o^ are predicted to play an important role in maintaining these in the preimplantation stage of development. Apart from single-copy endogenous cellular genes, retention of DNA methylation in the early embryo may help prevent retrotransposition of selfish DNA elements such as IAP, as these are transcriptionally derepressed in methyltransferase-deficient embryos [56], [57] and ES cells [58]. This could potentially lead to insertional mutagenesis and aberrant gene expression by transposon promoters inserted in or near genes [59]. In adult differentiated cells, alterations in DNMT1 levels can have different outcomes depending on degree of reduction and on the cell type affected. While none of our mutations or knockdowns characterised here completely blocked DNMT1 translation, deletion of the entire conserved region reduced translation of a reporter to approximately 25% in both ES and adult cells, as did stable knockdown of CPEB1 in HeLa. While we observed no changes in cell viability in the HeLa cells, which are transformed, comparable reductions in DNMT1 levels in a normal, untransformed cell line triggered the DNA damage response, decreased cell viability and affected DNA repair capabilities [10]. Reductions in DNMT1 are also associated with pathological conditions such as aggressive T-cell lymphoma [60]. Interestingly, mice expressing reduced but not abolished levels of DNMT1 specifically in post-mitotic neurons showed resistance to the effects from ischemic brain injury [61].

In summary, our work indicates that the *Dnmt1* mRNA is subject to combinatorial control by two established regulatory factors, CPEB1 and Musashi1, during oogenesis and in proliferating somatic cells. In both cases, these factors appear to promote *Dnmt1* translation. A greater understanding of the components involved in the regulation of *Dnmt1* mRNA translation will further our insights into embryonic development and disease, where DNMT1 plays an important role.

## Supporting Information

Table S1 **Gene specific primers for poly(A) analysis.**
(DOCX)

Table S2 **Primers and annealing temperatures for RT-PCR.**
(DOCX)

Table S3 **Primers for RT-qPCR.**
(DOCX)

Table S4 **Primary and secondary antibodies for Western analysis.**
(DOCX)

Table S5 **RIP Primers and annealing temperatures for RT-PCR and RT-qPCR.**
(DOCX)
