# Supplementary material for: Efficient Translation of Dnmt1 Requires Cytoplasmic Polyadenylation and Musashi Binding Elements
Source: PLoS One. 2014 Feb 20;9(2):e88385. doi: 10.1371/journal.pone.0088385 (PMC3930535; doi:10.1371/journal.pone.0088385)
Supplement: Table S1 — Gene specific primers for poly(A) analysis. (DOCX) [file pone.0088385.s001.docx]

**Table S1. Gene specific primers for poly(A) analysis**

| **Gene** | **Primer (Forward)** |
| --- | --- |
| *Gdf9* (m) | 5’- ATGATAGCTACGAGGTGCACC -3’ |
| *Dnmt1* (m) | 5’-CCATCATTTGAAGTCTTGTGC-3’ |
| *Dnmt1* (X) | 5’-GGATTTTATACGTTGTTACG -3’ |
