# Supplementary material for: Efficient Translation of Dnmt1 Requires Cytoplasmic Polyadenylation and Musashi Binding Elements
Source: PLoS One. 2014 Feb 20;9(2):e88385. doi: 10.1371/journal.pone.0088385 (PMC3930535; doi:10.1371/journal.pone.0088385)
Supplement: Table S2 — Primers and annealing temperatures for RT-PCR. (DOCX) [file pone.0088385.s002.docx]

**Table S2. Primers and annealing temperatures for RT-PCR**

| **Gene** | **Primers** | **Annealing**  **Temp (°C)** |
| --- | --- | --- |
| *Cpeb1* (m) | F 5’-ATGGACCAAGAACAAGCTGC-3’  R 5’-TCATGAGAACAAGCCTGAAGC-3’ | 58°C |
| *CPEB1* (h) | F 5’-GTATGTGTATCTGGTCTTCG-3’  R 5’- AAGTCACACGACCAGAACC-3’ | 56°C |
| *Msi1* | F 5’-TCACTTTCATGGACCAGGCG–3’  R 5’–TAACTCGGGCTGGCGTAGGT– 3’ | 56°C |
| *Actb* (m) | Obata and Kono, 2002 | 56°C |
| *ACTB* (h) | F 5’- CGTGGACATCCGCAAAGACC-3’  R 5’- TGTTTTCTGCGCAAGTTAGG-3’ | 56°C |
| *Luciferase* | F 5'-CATAGAACTGCCTGCGTGAG-3'  R 5'-CCCCGACTTCCTTAGAGAG-3' | 56°C |
| *Renilla* | F 5'-ATCTACGTGCAAGTGATGATTTACC-3'  R 5'-TTGTTGTTAACTTGTTTATTGCAGC-3' | 56°C |
