# Supplementary material for: Efficient Translation of Dnmt1 Requires Cytoplasmic Polyadenylation and Musashi Binding Elements
Source: PLoS One. 2014 Feb 20;9(2):e88385. doi: 10.1371/journal.pone.0088385 (PMC3930535; doi:10.1371/journal.pone.0088385)
Supplement: Table S3 — Primers for RT-qPCR. (DOCX) [file pone.0088385.s003.docx]

**Table S3. Primers for RT-qPCR**

| **Gene** | **Primers** |
| --- | --- |
| *CPEB1* | F 5’- CCTGGGTATTAGCCGACAGT -3’ |
|  | R 5’- GCCTCAGCATTTAGCATTCC -3’ |
| *DNMT1* | F 5’- GTGGGGGACTGTGTCTCTGT - 3’ |
|  | R 5’- TGAAAGCTGCATGTCCTCAC - 3’ |
| *HPRT* | F 5’- AGCCCTGGCGTCGTGATTAGT - 3’ |
|  | R 5’- CCCCTTGAGCACACAGAGGGCTA - 3’ |
| *ACTB* | F 5’- GGACTTCGAGCAAGAGATGG -3’ |
|  | R 5’- AGCACTGTGTTGGCGTACAG - 3’ |
| *Msi1* (m) | F 5’-CAGGCACAGAGGGTTTGGAT–3’ |
|  | R 5’–GTAGGTCGTGGCTTGGAACC– 3’ |
| *Msi2* (m) | F 5’-GGCAGACCTCACCAGATAGC–3’ |
|  | R 5’–TGACCATCTTAGGTTGCGCT– 3’ |
| *Hprt* (m) | F 5’–GGAGATGATCTCTCTCAACTTT– 3’ |
|  | R 5’–CCAACAACAACTTGTCTGG– 3’ |
