# Supplementary material for: Efficient Translation of Dnmt1 Requires Cytoplasmic Polyadenylation and Musashi Binding Elements
Source: PLoS One. 2014 Feb 20;9(2):e88385. doi: 10.1371/journal.pone.0088385 (PMC3930535; doi:10.1371/journal.pone.0088385)
Supplement: Table S4 — Primary and secondary antibodies for Western analysis. (DOCX) [file pone.0088385.s004.docx]

**Table S4. Primary and secondary antibodies for Western analysis**

| **Protein** | **Primary Antibody** | **Secondary antibody** |
| --- | --- | --- |
| CCNB1 | H433; Santa Cruz (Heidelberg, Germany); 1:300 | Goat anti-rabbit  (sc-2004); Santa Cruz; 1:10000 |
| CPEB1 | K16; Santa Cruz; 1:500 | Donkey anti-goat  (sc-2020); Santa Cruz; 1:5000 |
| DNMT1 | ab13537; Abcam (Cambridge, UK); 1:500 | Rabbit anti-mouse (A9044); Sigma-Aldrich; 1:5000 |
| GAPDH | 14C10; Cell Signalling Technology (Hitchin, UK); 1:10000 | Goat anti-rabbit (see above) |
| DNMT1 (m) | antigen region amino acids 331-647 (1:2000) | Goat anti-rabbit (see above) |
| B-ACTIN | A1978; Sigma (UK); 1:500 | Rabbit anti-mouse (see above) |
