# Supplementary material for: Efficient Translation of Dnmt1 Requires Cytoplasmic Polyadenylation and Musashi Binding Elements
Source: PLoS One. 2014 Feb 20;9(2):e88385. doi: 10.1371/journal.pone.0088385 (PMC3930535; doi:10.1371/journal.pone.0088385)
Supplement: Table S5 — RIP Primers and annealing temperatures for RT-PCR and RT-qPCR. (DOCX) [file pone.0088385.s005.docx]

**Table S5. RIP Primers and annealing temperatures for RT-PCR and RT-qPCR**

| **Gene** | **Primers** | **Annealing** |
| --- | --- | --- |
|  |  | **Temp (°C)** |
| *Dnmt1* (m) | F 5’- GGCTTTCCAGATAGCTACCG -3’ | 58°C |
|  | R 5’- GCAGGCAGAGCTTAATCTCC -3’ |  |
| *Numb* (m) | F 5’- AACACAGTGCCCGAGGTGGAAG -3’ | 54°C |
|  | R 5’- ATGGGAGGCTGAGTCAGTGCCA -3’ |  |
| *Hprt* (m) | F 5’- GGAGATGATCTCTCAACTTT -3’ | 60°C |
|  | R 5’- CCAACAACAAACTTGTCTGG -3’ |  |
